# Colonic perforation secondary to post endoscopic retrograde cholangio-pancreatography severe pancreatitis: a case report on the lethal potential of neglected cholelithiasis with review of literature

**DOI:** 10.1093/jscr/rjag133

**Published:** 2026-03-07

**Authors:** Md Anas, Imad Ali, Hazique Jameel, Shivendu Purushottam

**Affiliations:** Department of Surgery, Jawaharlal Nehru Medical College, AMU, Aligarh 202001, India; Department of Surgery, Jawaharlal Nehru Medical College, AMU, Aligarh 202001, India; Department of Surgery, Jawaharlal Nehru Medical College, AMU, Aligarh 202001, India; Department of Surgery, Jawaharlal Nehru Medical College, AMU, Aligarh 202001, India

**Keywords:** colonic perforation, pancreatitis, endoscopic retrograde cholangiopancreatography (ERCP), cholelithiasis

## Abstract

A 40-year-old lady with a known history of gallstones presented with acute abdominal pain. Diagnostic imaging confirmed cholelithiasis with concomitant choledocholithiasis. She underwent a therapeutic endoscopic retrograde cholangiopancreatography for stone extraction. Her post-procedural course was complicated by the development of severe acute pancreatitis. Despite aggressive medical management, her clinical condition deteriorated rapidly. Subsequent computed tomography scans revealed extensive pancreatic necrosis and a perforation of the splenic flexure of the colon. The patient was taken for emergency surgery. An exploratory laparotomy was performed. Resection of the affected splenic flexure, a primary anastomosis, and the creation of a proximal loop ileostomy done. The patient recovered without further major complications. She was discharged in satisfactory condition with plans for ileostomy reversal later on. This case underscores the dictum that in medicine, a benign-looking pathology can have malignant consequences, demanding respect, timely intervention, and relentless monitoring.

## Introduction

Cholelithiasis or gallstone disease, affects a significant portion of the adult population. While often asymptomatic, it can lead to severe complications such as acute cholecystitis, cholangitis, and pancreatitis if untreated [1].

Endoscopic retrograde cholangiopancreatography (ERCP) is a widely used minimally invasive procedure for diagnosing and treating Choledocholithiasis, the presence of gallstones in the common bile duct (CBD) [2].

A well-documented complication of ERCP is post-ERCP pancreatitis, occurring in 3%–10% of cases. Most cases are mild and self-limiting. However, a small subset of patients develops severe, necrotizing pancreatitis, which carries substantial morbidity and mortality. Colonic complications of severe pancreatitis including ischemia, fistula formation, and perforation are rare but catastrophic, resulting from the direct enzymatic action of pancreatic exudates and local vascular compromise [2].

This case report describes a rare escalation of events in a 40-year-old lady where post-ERCP severe acute pancreatitis resulted in colonic perforation, necessitating emergency surgery. It serves as a cautionary example of how seemingly ‘simple’ gallstones can become life-threatening if ignored or if procedural complications arise, emphasizing the need for early intervention and vigilant monitoring.

## Case report

A 40-year lady presented to emergency with complaints of severe pain abdomen and vomiting. She was already diagnosed case of symptomatic cholelithiasis with choledocholithiasis at an outside facility, for which endoscopic retrograde cholangiopancreatography (ERCP) with sphincterotomy with pancreatic duct (PD) and CBD stenting was done 10 days back. Since her clinical condition was not improving. So, she presented to our hospital for more intensive treatment.

### Examination

On presentation her vitals were temperature 38.0°C, heart rate 122 bpm, respiratory rate 26, blood pressure 104/62 mm Hg @ Norad 4 ml/hr, and oxygen saturation 91% on room air. She was in acute distress. Abdominal examination showed distended abdomen, diffuse tenderness without rigidity and decreased bowel sounds.

### Laboratory tests

Haemoglobin 9.2 g/dl; WBC 17200 cells/mm3 (neutrophils 75.3%); AST 118 IU/L; ALT 123 IU/L; alkaline phosphatase 231 IU/L; total bilirubin 0.5 mg/dl; serum amylase 817 U/L; serum lipase 1840 U/L; and serum lactate 3.9 mmol/L, blood urea nitrogen 26 mg/dl, creatinine 1.9 mg/dl.

## Imaging

**Figure 1:**
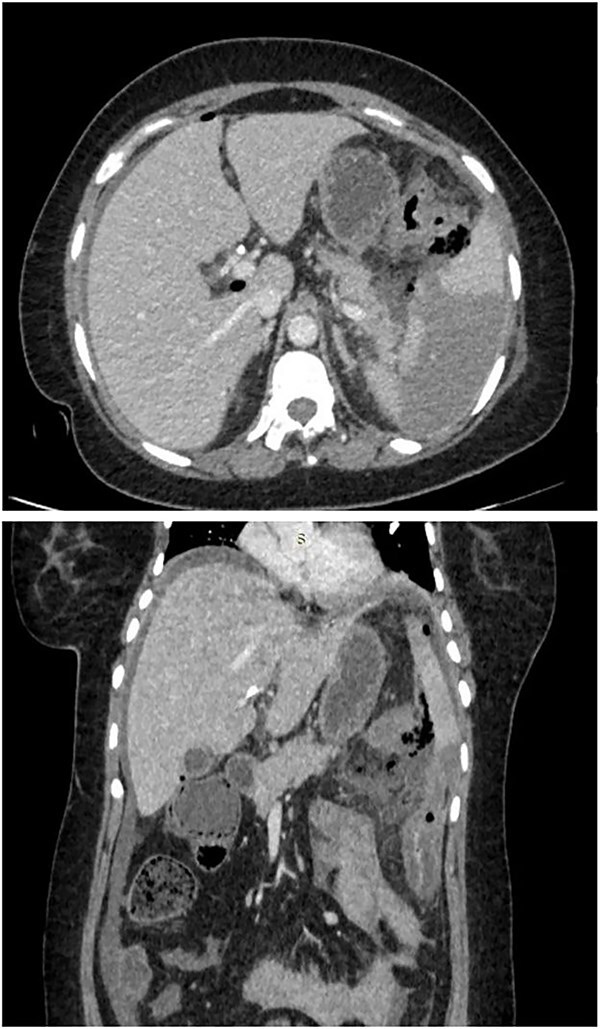
CT scan of abdomen showed pneumoperitoneum in inter bowel space, pancreatic necrosis, peripancreatic collection in splenic flexure region with multiple air foci within it and adjacent mesenteric fat stranding and heterogeneity. (? Splenic flexure of colon perforation).

As shown in Fig. 1, computed tomography (CT) scan of abdomen showed pneumoperitoneum in inter bowel space, pancreatic necrosis, peripancreatic collection in splenic flexure region with multiple air foci within it and adjacent mesenteric fat stranding and heterogeneity. (? Splenic flexure of Colon perforation).

### Management

**Figure 2:**
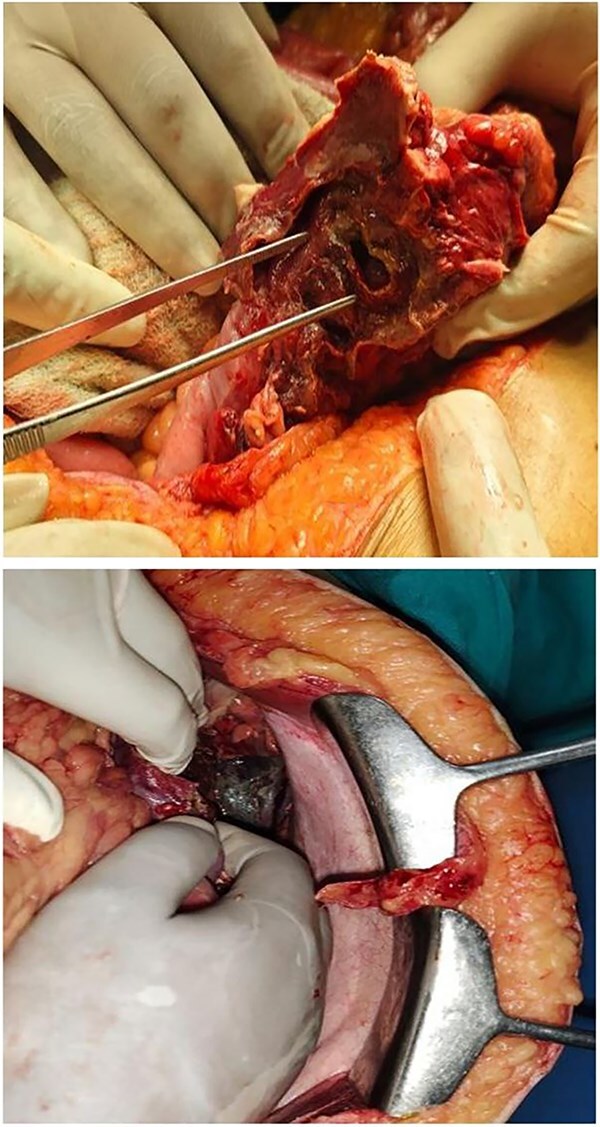
2 × 1 cm splenic flexure of colon perforation & necrotic pancreas.

Patient was taken for emergency laparotomy which showed ~200 ml of pancreatic free fluid with purulent and feculent material, calcified pancreas with dense adhesions, 2 × 1 cm perforation in splenic flexure of colon as shown in Fig. 2. Resection and anastomosis of splenic flexure of colon with proximal loop ileostomy with placement of 2 abdominal drain was done. Patient was shifted to intensive care unit for post op care.

### Outcome

The post op period was uneventful. Patient was discharged in stable condition after 2 weeks of hospital stay. She continued to follow-up in the outpatient department and is currently doing well. Now, she is planned for ileostomy closure soon.

## Discussion

Colonic complications are observed in up to 10% of patients diagnosed with severe acute pancreatitis and include ileus, abscess, obstruction, fistula, and perforation [3]. Colonic perforation is an infrequent medical event, and as such, the available literature consists primarily of case studies.

The patient in this report experienced a perforation at the splenic flexure of colon and it is also the most common site. The mechanism of colonic perforation in acute pancreatitis is multifactorial. The close anatomical relationship between the pancreatic tail and the splenic flexure of the colon makes it vulnerable. The release of proteolytic pancreatic enzymes can lead to direct digestion of the colonic wall. Furthermore, the intense inflammatory response can cause thrombosis of mesenteric vessels, leading to ischemic necrosis and subsequent perforation. The splenic flexure is particularly susceptible due to its watershed blood supply [4].

Research from Nakanishi *et al*. provides some of the limited data, indicating a 5.3% incidence of colon perforation with a median onset of 13 days following the start of symptoms. The timing of this patient's colonic perforation, at 10 days after the initial symptoms of acute pancreatitis, is consistent with the median onset time observed in other reported cases [5].

The mortality rate for severe acute pancreatitis can be as high as 30%. When surgical treatment is required, this risk escalates to ~40% because of the additional stress. As described in Table 1, similar mortality reported in many cases by Liew [3], Nagpal [4], and Nisserine *et al.* [6].

**Table 1:** Summary of major studies on colonic perforation with pancreatitis

Author / Year	Age / Sex	Site	Cause of pancreatitis	Procedure	Outcome
Liew *et al.*, 2025 [3]	61y/ Female	Caecum	Idiopathic	Exploratory laparotomy and oversown perforation	Uneventful
	72y/ Male	Sigmoid colon	Gallstones	Total colectomy	Uneventful
Nissrine *et al.*, 2025 [6]	38y / Male	Transverse colon	Gallstones	Exploratory laparotomy	Intraoperative death
Saleh *et al.*, 2024 [7]	60y / Female	Sigmoid colon	Idiopathic	Exploratory laparotomy with diversion colostomy	Uneventful
Hozaka *et al.*, 2019 [8]	31y / Male	Splenic flexure of colon	Gallstones	Video-assisted retroperitoneal debridement with proximal loop ileostomy	Uneventful
Dhadlie *et al.*, 2019 [9]	70y / Male	Ascending colon	Gallstones	Exploratory laparotomy with right sided hemicolectomy with end ileostomy	Uneventful
Nagpal *et al.*, 2015 [4]	35 y / Male	Splenic flexure of colon	Gallstones	Exploratory laparotomy with subtotal colectomy with proximal loop Ileostomy	Uneventful
	54y / Male	Transverse colon	Gallstones	Exploratory laparotomy with subtotal colectomy with proximal loop Ileostomy	Death in post operative period
Naidoo *et al.*, 2014 [2]	41y / Female	Transverse colon	Post ERCP	Extended right hemicolectomy with end-ileostomy	Uneventful

These patients are often septic, even when receiving the highest level of conservative care, indicating a need for urgent surgical management. Source control through colonic resection and damage control surgery is crucial for immediate sepsis resolution [3]. Furthermore, to reduce the risk of anastomotic leaks, a diverting loop ileostomy is recommended if an anastomosis is performed, as it reduces the overall hospital stay and mortality. Similar result found in our case, 2 weeks stay as compared to longer stay (1 month – 3 month) in other reports [4].

## Conclusion

Neglected symptomatic gallstone disease is not benign. It can set off a cascade of severe, life-threatening complications. This case report serves as a stark reminder that severe post-ERCP pancreatitis can lead to rare but devastating extra pancreatic consequences like colonic perforation. It emphasizes the importance of timely cholecystectomy in patients with symptomatic gallstones to prevent the need for higher-risk procedures and their potential complications. This case underscores the dictum that in medicine, a benign-looking pathology can have malignant consequences, demanding respect, timely intervention, and relentless monitoring.
